# Herb-Induced Liver Injury by *Laurus nobilis*: A Case Assessed for Causality Using the Updated RUCAM

**DOI:** 10.3390/life16010180

**Published:** 2026-01-22

**Authors:** Mihnea Soare, Sabina-Florina Călugăr-Șolea, Ciprian Brisc, Marius Rus, Teodora-Maria Bodog, Gabriel Becheanu, Ciprian Mihai Brisc, Mihaela-Cristina Brisc

**Affiliations:** 1 Doctoral School of Biological and Biomedical Sciences, University of Oradea, 410087 Oradea, Romania; soare.mihneavlad@student.uoradea.ro (M.S.); solea.sabinaflorina@student.uoradea.ro (S.-F.C.-Ș.); bodog.teodoramaria@student.uoradea.ro (T.-M.B.); brisccristina@uoradea.ro (M.-C.B.); 2 Department of Medical Disciplines, Faculty of Medicine and Pharmacy, University of Oradea, 410073 Oradea, Romania; 3 Department of Surgical Disciplines, Faculty of Medicine and Pharmacy, University of Oradea, 410073 Oradea, Romania; 4 National Institute of Pathology and Biomedical Sciences Field “Victor Babeș”, 050096 Bucharest, Romania; gabriel.becheanu@umfcd.ro; 5 Medicine Program of Study, Faculty of Medicine and Pharmacy, University of Oradea, 410073 Oradea, Romania; brisc.ciprianmihai@student.uoradea.ro

**Keywords:** HILI, *Laurus nobilis*, hepatotoxicity, updated RUCAM

## Abstract

Hepatocellular injury syndrome represents a pathological process with a broad etiological spectrum, including viral infections, autoimmune diseases, or intoxications. Clinicians must identify the potential cause using both anamnestic data and available paraclinical examinations. We present the case of a 55-year-old female patient, admitted to the Internal Medicine 1 Department at the Clinical County Emergency Hospital Bihor, Oradea, Romania. The patient exhibited nonspecific complaints and insignificant pathological antecedents, but from a biochemical perspective, substantial changes in liver transaminase levels were evident. To establish differential diagnoses, a series of biochemical and immunological tests were performed, along with a thorough medical history. It was concluded that the patient regularly consumes herbal infusions, specifically *Laurus nobilis* leaves, commonly known as Bay Laurel. Although this might be easily overlooked at first glance, a closer examination could explain the current clinical picture. In April 2024, a 55-year-old female patient with no history of liver pathology was admitted. She complained of asthenia fatigue, anorexia, mixed dyspeptic symptoms, diffuse abdominal pain, and a weight loss of 12 kg. The pathology had insidiously started approximately 3 months prior. On examination, the patient had altered general status, anorexia, and was overweight. Biochemically, the patient had elevated liver transaminase values (AST = 196 U/L and ALT = 357 U/L) that continued to rise during hospitalization, despite hepatoprotective treatment. Various paraclinical examinations were performed to exclude other potential causes of hepatic aggression, having excluded ordinary causes. Consequently, a liver biopsy was performed, and the histopathological examination leaned toward a toxic hepatitis etiology. Application of the updated RUCAM scale yielded a score of eight points (“probable” HILI—Herb-Induced Liver Injury). Clinical and biochemical improvement was observed after complete cessation of bay leaf tea consumption. This case highlights the potential hepatotoxicity of commonly used culinary herbs when consumed in large quantities or as concentrated infusions and emphasizes the importance of detailed anamnesis regarding herbal product use.

## 1. Introduction

Hepatocellular injury syndrome represents a pathological process with a wide etiological spectrum, including viral infections, autoimmune diseases, and intoxications. The clinician must identify the underlying cause using both anamnestic data and available paraclinical investigations. In certain clinical situations, the etiology of the hepatic injury remains elusive despite the use of standard diagnostic algorithms and advanced paraclinical workups. In such cases, the evaluation must be expanded through an exhaustive anamnesis, focusing on discrete but potentially relevant factors, such as self-medication, chronic consumption of phytotherapeutic supplements, or exposure to substances with hepatotoxic potential, which may constitute possible causes of liver injury [1,2,3]. Drug-Induced Liver Injury (DILI) is a pathological entity characterized by liver damage mediated by xenobiotic compounds of a medicinal type, occurring in the context of pharmacological exposure and in the absence of any other identifiable alternative etiology [2,3]. It is classified into predictable forms, with a toxic–metabolic mechanism that is dose-dependent, and idiosyncratic forms, which are dose-independent and have an immunological or genetic substrate [2,3]. Herb-Induced Liver Injury (HILI) represents a subcategory of DILI, in which the liver pathology is associated with the use of plant-based products [1,2]. The interest in alternative therapies based on natural remedies, involving the use of herbal extracts or medicinal plants with high accessibility and widespread presence in popular culture, is a frequently encountered practice among the general population [1]. These are often administered either by virtue of empirically transmitted ethnomedical traditions or as a result of recommendations from non-specialized sources, particularly from the online environment [1].

## 2. Case Presentation

In April 2024, patient S.L., a 55-year-old female with no prior documented liver disease, was admitted to the 1st Department of Internal Medicine of the Clinical County Emergency Hospital Bihor, presenting with a constitutional syndrome of progressive onset over approximately three months, characterized by asthenia and fatigue, anorexia, mixed dyspeptic syndrome, abdominal discomfort, and unintentional weight loss of approximately 12 kg, with symptom exacerbation in the days immediately preceding admission. Physical examination revealed the following: an overweight nutritional status (BMI 28.03 kg/m^2^) with a height of 170 cm and weight of 81 kg; clear skin, without any vesicular-type eruption, conjunctiva, mucosa, or color modifications; uncharacteristic face; no enlargement of the superficial lymph nodes; waist circumference of 88, normal conformation of the thorax; no pulmonary rales; BP of 138/85 mmHg (under medication, with no episodes of hypotension); rhythmic pulse, 68 b/min; no cardiac murmurs; an aspect of the abdomen slightly enlarged due to abdominal adiposity, without abdominal tenderness; absence of acute abdomen signs; normal digestive transit for stool and gas; liver was palpable immediately under the costal rim, with a smooth surface and round inferior border, with normal consistency and no liver pain at palpation; and the spleen not accessible for palpation. The patient reported no modification of the color of the urine and stool. From the clinical point of view, there were no modifications at the level of renal system and urinary tract. Personal physiological and pathological history included two cesarean deliveries, menopause onset at age 46, and essential arterial hypertension treated with combined antihypertensive therapy (Perindopril/Indapamide/Amlodipine 5 mg/1.25 mg/5 mg, 1 tablet/day, and Bisoprolol 2.5 mg/day), medication with no potential hepatic toxicity. She denied personal or family liver disease history. From a socio-occupational perspective, the patient is employed as auxiliary cleaning and sanitation staff, without direct occupational exposure to potentially hepatotoxic agents. She resides in an urban environment (Oradea) with adequate living conditions. Anamnestic, the patient reports occasional coffee consumption and denies alcohol intake (religiously motivated). However, our patient reported regular consumption over the preceding three months of infusions prepared from bay leaves (*Laurus nobilis*), which she had initiated on her own initiative following information obtained from publicly available online sources. The intake was described as motivated by perceived health-related (“detoxification”) benefits. According to Herb Medicine Indonesia, five dried bay leaves weigh approximately 1–1.5 g, corresponding to an estimated individual leaf mass of 0.2–0.3 g [4]. Based on the patient’s description, each infusion was prepared using one to two crushed dried bay leaves and was consumed twice daily, corresponding to an estimated daily intake of approximately 0.8–1.2 g of dried bay leaf and to a dose of 9.8–14 milligrams/kilogram of Body Weight/day.

At admission, laboratory findings showed hepato-cytolytic injury syndrome (AST = 196 U/L and ALT = 261 U/L), dyslipidemia (LDL cholesterol = 120.24 mg/dL, HDL cholesterol = 39 mg/dL), and an impaired fasting glucose level (119 mg/dL) (Table 1). Abdominal ultrasound revealed steatosis grade II, and aspect and abdominopelvic CT revealed mesenteric panniculitis accompanied by inflammatory-appearing lymphadenopathy. Both imagistic methods demonstrated the normal size of the liver and of the spleen. Transient elastography (FibroScan) showed the following values: CAP = 265 dB/m—steatosis grade S0 (cut-off value S0/S1 = 288 dB/m); fibrosis − E = 3.5 kPa equivalent to Metavir F0/F1. To exclude possible viral hepatic cytolysis, serology for hepatitis A (IgM VHA), B (HBsAg and total anti-HBc IgG+IgM), C (Ac HCV), and E (IgM+IgG VHE) viruses were performed, with negative results. Serology also excluded possible hepatitis due to Epstein–Barr virus and cytomegalovirus. An autoimmune hepatopathy was considered; thus, an autoimmune hepatitis panel was performed. Anti-mitochondrial M2 antibodies (AMA-M2) and anti-M2-3E were assayed to rule out primary biliary cholangitis (results within normal limits), antinuclear antibodies (ANAs) to exclude autoimmune hepatitis (AIH) type 1, anti-liver-kidney microsomal type 1 antibodies (LKM-1) and anti-liver cytosol type 1 (anti-LC1) for differential diagnosis with AIH type 2, and anti-soluble liver antigen/liver-pancreas (anti-SLA/LP) for AIH type 3, as well as other antibodies specific to autoimmune liver diseases (anti-sp100, anti-PML, anti-gp210, and anti-Ro-52), all of which were negative. To exclude hereditary liver disease, ceruloplasmin (Wilson’s disease) and alpha-1 antitrypsin levels were measured, with results within normal limits.

The liver coagulation tests were normal, and electrophoresis of proteins revealed an albumin serum level of 4.19 g/dL, (normal range 4.02–4.76 g/dL), a normal range of alpha- and beta-globulins, and a serum level of gamma globulins of 1.29 g/dL (normal range 0.80–1.35 g/dL)

Also, we verified the presence of Helicobacter Pylori Antigen in feces and we checked hemoccult tests, both results being negative.

During the first hospitalization on 5–18 April 2024, the patient received hepatoprotective therapy consisting of silymarin, essential phospholipids and amino-acids, and L-arginine, alongside solutions for metabolic support and hydro-electrolytic rebalancing. Initially favorable clinical evolution allowed discharge according to the patient’s choice, although the hepatocellular injury syndrome was still present. We recommended the cessation of any dietary habits she adopted lately and treatment with oral administration of arginine and silymarin. However, the patient was re-admitted in the hospital after two weeks (2 May 2024), due to symptom recurrence. In accordance with the clinical status, the laboratory findings were concerning as well, as the transaminases values had increased again, even though our patient had followed our advice both in terms of pharmacological treatment and the cessation of infusion intake.

**Figure 1:**
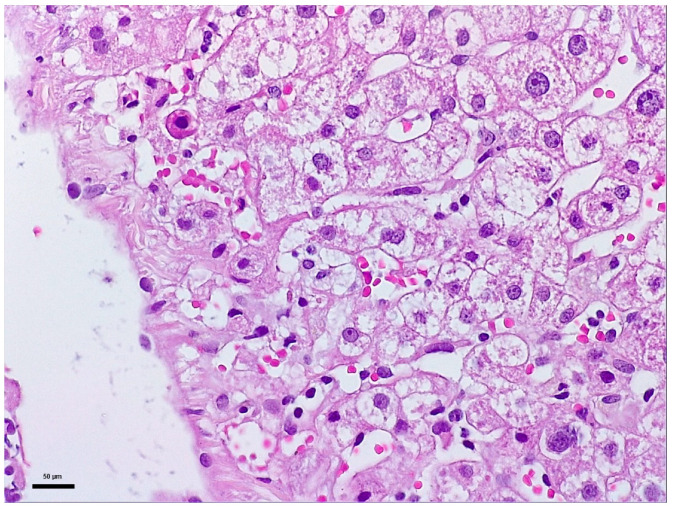
Biopsy fragment 1: Representative histological image of liver tissue stained with Hematoxylin and Eosin (H&E) staining, observed under a 20× objective (total magnification 200×). Scale bar: 50 μm.

**Figure 2:**
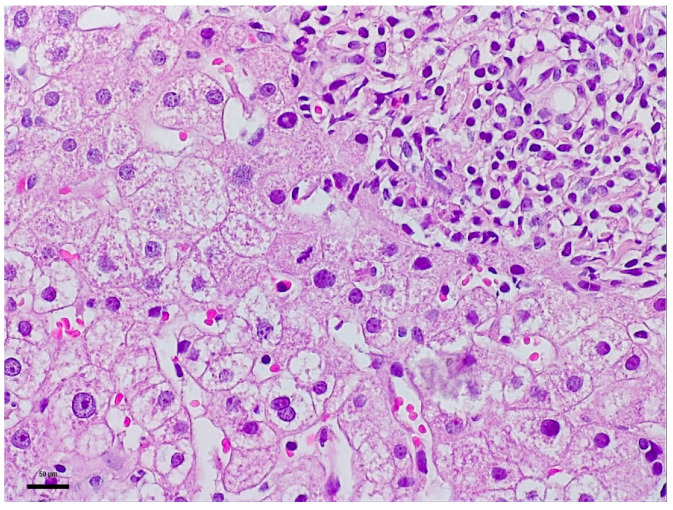
Biopsy fragment 2: Representative histological image of liver tissue stained with Hematoxylin and Eosin (H&E) staining, observed under a 20× objective (total magnification 200×). Scale bar: 50 μm.

To elucidate the etiopathogenesis of the hepatocellular injury syndrome, a liver biopsy with Bard Magnum True-Cut 18 G was performed during the patient’s second hospital admission. This liver biopsy proceeded without complications or incidents and under full safety conditions. Histology described mild-to-moderate portal tract expansion with lympho-plasmocytic inflammation and a preserved portal triad without pathological fibrosis and without interface hepatitis. Numerous apoptotic hepatocytes and focal confluent necrosis were noted but without bridging necrosis. No histological signs of cholestasis or severe steatosis were identified. The overall picture was that of moderate-to-severe portal and lobular hepatitis without specific features; therefore, a possible herb-induced liver injury (HILI) could not be excluded (Figure 1 and Figure 2).

“The biopsy core measures 14 mm in length and, on histological sectns, is thin but adequate, showing preserved lobular architecture of the hepatic parenchyma. Two portal tracts and seven centrilobular veins are identified. The portal tracts are mild to moderately expanded with lymphoplasmacytic inflammatory infiltrate; the portal triad is preserved, without pathological fibrosis and without interface hepatitis. Within the lobules, there is hepatocellular ballooning (dystrophic change), numerous apoptotic hepatocytes, focal and confluent necrosis without bridging necrosis; frequent typical mitoses and regenerative hepatocellular rosettes are also noted. There is no histological evidence of cholestasis or pathological steatosis.” The histopathological interpretation corresponds to Figure 1 and Figure 2.

During the second hospitalization, the therapeutic strategy previously instituted at the initial admission was re-implemented. An updated RUCAM assessment was adopted, providing a total score of eight points, enough to sustain the following diagnostic: probable herb-induced liver injury. The management approach, in terms of medication and dietary advice, was associated with a marked improvement in both clinical status and biochemical parameters, permitting patient discharge on 13 May 2024. Subsequently, the patient was maintained under medical surveillance and definitively ceased any form of bay leaf consumption.

**Figure 3:**
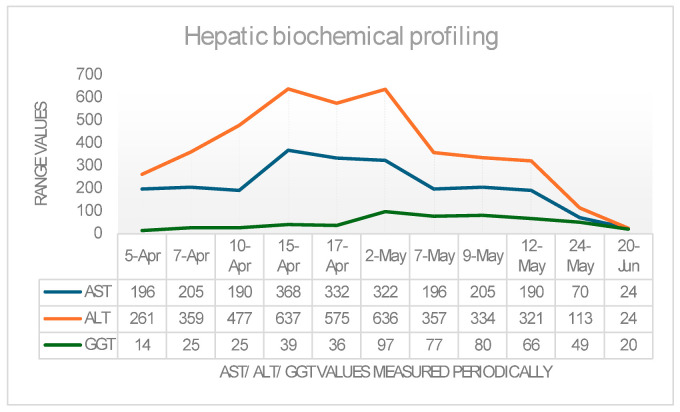
Evolution of the hepatic biochemical profile.

**Figure 4:**
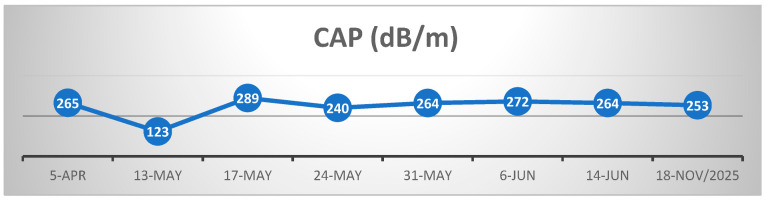
Evolution of CAP (controlled attenuation parameter) values during FibroScan monitoring.

**Figure 5:**
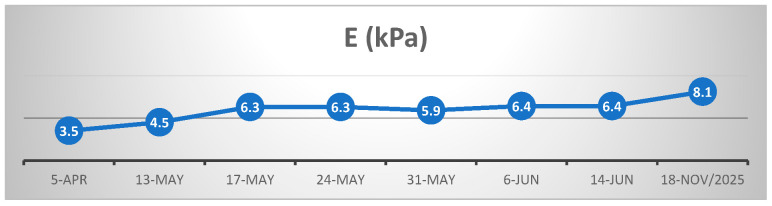
Evolution of E (liver stiffness) values during FibroScan monitoring.

For a better understanding of the disease’s evolution and progression, we added Figure 3 below, which includes the biologic monitorization (transaminases and GGT) and the progression of the parameters measured by the FibroScan. CAP (controlled attenuation parameter) and liver stiffness values during monitoring are detailed in Figure 4 and Figure 5.

In Figure 3 an upward trend in liver enzymes can be observed, which continued even after the first hospitalization, reaching a maximum peak in early May, shortly before the second hospitalization. Subsequently, under continuous hepatoprotective treatment, a rapid downward trend followed, with transaminases and GGT levels returning to normal values in the third decade of June of the same year.

Figure 4 shows the evolution of the CAP obtained by serial measurements using FibroScan, the mean value corresponding to a mild steatosis, in total accord with the ultrasound examination we performed in the first hospitalization and with the histological results, which provided no signs of pathologic steatosis. Despite the severity of clinical status, the values of the CAP did not vary in a large range, even after the critical period and recovery.

Longitudinal assessment of liver stiffness (Figure 5) demonstrated a persistent increase from the initial hospitalization, until maximum peak, in parallel with the highest elevations of transaminases and GGT (mid-April to early May), followed by a phase of relative stabilization. Notably, the final FibroScan evaluation in November 2025 revealed a further significant increase in liver stiffness despite minimal variation in the controlled attenuation parameter (CAP). This sustained upward trend of the liver stiffness (E) parameter suggests that the initial episode of extensive hepatocellular necrosis may have been followed by hepatic remodeling, potentially reflecting progression from absent or mild fibrosis (F0/F1) to significant fibrosis (F2) over an 18-month period. This hypothesis could not be histologically confirmed, as repeat liver biopsy was declined by the patient.

The patient’s clinical and biological evolution was slowly favorable following the discontinuation of bay leaf infusions, with the patient remaining under ongoing medical follow-up.

## 3. Discussion

*Laurus nobilis* and its extracts have been the subject of numerous medical and pharmaceutical studies. Its leaves contain various alkaloids, tannins, and flavonoids, with well-documented antimicrobial and antioxidant effects but also recognized hepatotoxic potential within the spectrum of herb-induced liver injury (HILI). Hepatotoxicity remains the primary adverse effect of many herbal products, and the diagnosis of HILI is often challenging because traditional diagnostic methods cannot precisely establish causality between the phytochemical agent and the hepatic-cytolysis syndrome. Bay laurel (*Laurus nobilis*) is a shrub from the Lauraceae family, commonly cultivated for ornamental purposes, with leaves widely used in gastronomy for their aromatic properties. The essential oil content in *Laurus nobilis* leaves ranges from 1% to 3%, depending on geographic origin and harvest period, and contains sesquiterpene lactones, with predominant compounds being 1,8-cineole (eucalyptol), α-pinene, β-pinene, α-terpinyl acetate, sabinen, linalool, and, in relatively low concentrations, eugenol and methyl-eugenol [5]. Among these, methyl-eugenol is considered the primary potentially hepatotoxic agent [6]. This aromatic compound (methyl-eugenol) undergoes hepatic metabolism via cytochrome P450 enzymes, yielding reactive intermediates such as 1′-hydroxymethyleugenol, which is genotoxic: it forms DNA adducts, induces oxidative stress, and triggers mitochondrial dysfunction, leading to cell necrosis and apoptosis via a p53-dependent pathway [7]. Notably, methyl-eugenol has been classified by the International Agency for Research on Cancer (IARC) as possibly carcinogenic to humans (Group 2B) based on preclinical studies demonstrating the development of hepatic neoplasms in rodents chronically exposed to high doses [8]. Eugenol, another constituent of bay leaf essential oils, is also recognized as a potential hepatotoxic agent. Acute eugenol overdose can result in severe liver injury characterized by acute hepatic necrosis, a clinical pattern closely resembling the hepatotoxicity induced by acetaminophen [9,10]. It is also well-recognized that bay laurel fruits (berries) are toxic. They contain significantly higher concentrations of aromatic oils compared with the leaves. Ingestion may cause acute intoxication with severe gastrointestinal symptoms.

In the context of this clinical case, application of the updated RUCAM (updated Roussel Uclaf Causality Assessment Method) score yielded eight points, indicating a probable causal relationship between repeated and sustained exposure to the suspected hepatotoxic agent and the observed liver injury [11]—see Table 2.

Herb-induced liver injury (HILI) is, to a large extent, a diagnosis of exclusion. Establishing this diagnosis requires an extensive and systematic evaluation aimed at ruling out other potential etiologies of liver injury. Accordingly, viral causes, autoimmune causes (based on the absence of specific serological markers and suggestive clinic and biological criteria), as well as genetic and metabolic disorders, were excluded. In addition, a careful assessment of exposure to other potentially hepatotoxic medications or toxic agents was performed, without identifying any plausible alternative cause. In this context, the temporal relationship between exposure and the onset of the hepatocellular injury pattern supports the diagnosis of HILI.

Based on the histopathologic description, the differential diagnosis appropriately excluded several entities like alcoholic liver disease, which typically shows steatohepatitis with cytoplasmic Mallory–Denk bodies, canalicular cholestasis, and characteristic “chicken-wire” perisinusoidal fibrosis [12]; metabolic dysfunction-associated fatty liver disease (MAFLD/MASH), characterized by macro-vesicular steatosis, hepatocyte ballooning, lobular inflammation, and perisinusoidal/portal fibrosis [13]; and autoimmune hepatitis, in which interface hepatitis (piecemeal necrosis) and/or dense portal lymphoplasmacytic infiltrate with periportal extension would be expected [14]. Thus, in the absence of specific histologic features and after exclusion of other common etiologies, herb-induced liver injury (HILI) secondary to chronic bay leaf infusion consumption remained the most plausible diagnosis. 

HILI may have a prolonged course, as herbal substances can contain multiple compounds with reactive metabolites and idiosyncratic or immune-mediated mechanisms. Variability in composition, chronic exposure, and delayed discontinuation of the offending agent favor persistence of liver injury even after withdrawal. The recovery period varies significantly depending on the type and severity of liver injury, the causative agent, and the patient’s pre-existing health status [15]. Nonspecific symptoms (fatigue and nausea) usually improve within a few weeks after discontinuation of the product; therefore, once clinical improvement was perceived, the patient did not continue clinical follow-up despite our recommendations. She only came for a FibroScan checkup in November 2025 but only in the Ambulatory of our Hospital; she did not want to be hospitalized to obtain biological samples because of her good general status.

Another noteworthy aspect is the idiosyncratic nature of the hepatotoxic reaction, given that the patient reported that other family members consumed the same *Laurus nobilis* leaf infusions without developing clinical signs suggestive of liver injury. This supports significant inter-individual variability in metabolism and hepatic response to the bioactive compounds contained therein.

Hepatocellular necrosis is a well-recognized consequence of xenobiotic exposure and is commonly driven by metabolic bioactivation, oxidative stress, and mitochondrial dysfunction. Classical toxicants such as paracetamol and certain non-steroidal anti-inflammatory drugs induce liver injury through cytochrome P450-mediated formation of some metabolites, along with a decrease in intracellular glutathione and trouble with mitochondrial metabolism, thus inducing apoptosis of the hepatocytes [16,17]. Polyphenols or constituents of essential oils, particularly eugenol-like phenylpropanoids, can undergo metabolic activation to electrophilic intermediates and promote redox imbalance, leading to mitochondrial dysfunction, oxidative stress, and glutathione disfunction when hepatic antioxidant defenses are exceeded [18,19]. Thus, these mechanisms can provoke hepatocyte death by depletion of ATP and membrane destabilization [20,21].

As the abdominal CT scan assessment revealed, in our patient there were present signs of mesenteric panniculitis. This was also a condition which concerned us related to hepatocellular injury, but the international literature we have studied [22,23,24] excluded this possibility. These papers revealed that in mesenteric panniculitis, the general inflammatory disorders are associated with cholestatic liver involvement in the male gender [21], which was not present in our patient and sometimes, it can be found in people with underlying malignancy, abdominal surgery or trauma, gallstones, or vasculitis [24]. However, our female patient did not have, nor developed, any of these diseases.

A similar case to ours was reported in the medical literature in 2023 from Turkey, in which a woman consumed *Laurus nobilis* leaf infusions for approximately one month. Subsequently, she developed symptoms suggestive of acute liver failure, including progressive asthenia, jaundice, abdominal pain, and marked elevation of hepatocellular injury and cholestasis markers. Investigations confirmed acute liver failure; despite initiation of specific treatment and planning for liver transplantation, the patient’s condition rapidly deteriorated, and death occurred before the procedure could be performed [10].

A further fatal case implicating the hepatotoxic potential of *Laurus nobilis* was reported in 2021 [25]. In that report, a 51-year-old woman developed acute fulminant liver failure following self-administration of bay laurel infusions, ultimately resulting in a fatal outcome.

Additional evidence supporting the potential hepatotoxicity of *Laurus nobilis* was reported earlier, in 2019, in a case involving a 64-year-old male patient who had ingested a herbal preparation containing Melissa officinalis, Hypericum perforatum, and *Laurus nobilis* three times daily for one week [26]. The patient presented a clinical picture consistent with acute liver failure, requiring admission to the intensive care unit. Owing to timely hospital presentation and appropriate supportive management, the patient survived.

A comprehensive systematic review published in 2024, encompassing 101 articles addressing drug-induced and herb-induced liver injury (DILI/HILI), updated the spectrum of herbal products with documented or suspected hepatotoxic potential worldwide [27]. The review identified numerous agents, including pyrrolizidine alkaloids, Aloe vera, Withania somnifera (ashwagandha), Psoralea corylifolia (Bakuchi), turmeric/curcumin, kratom, black cohosh, Morinda citrifolia (Noni), Senna alexandrina (Indian senna), alkaline water, and elderberry. Notably, despite the breadth of herb products analyzed, no specific association between *Laurus nobilis* and hepatotoxicity was identified in this extensive review [27].

Specific cases of herb-induced liver injury (HILI) related to herbal products other than *Laurus nobilis* have been documented, in which the RUCAM score was applied to support diagnostic attribution. One illustrative case describes a 63-year-old woman who developed severe acute hepatocellular hepatitis after ingestion of a concentrated green tea extract (Camellia sinensis). Thorough evaluation excluded viral-, autoimmune-, metabolic-, and drug-related etiologies. Causality assessment using the RUCAM method indicated a probable association between Camellia sinensis exposure and the observed liver injury [28]. Similarly, herb-induced liver injury has been reported following the use of Ayurvedic Withania somnifera (ashwagandha) supplements. Two cases involved adults who developed hepatocellular and mixed liver injury after prolonged intake of Withania somnifera. In both patients, comprehensive investigations ruled out alternative causes, and application of the updated RUCAM scale yielded scores of seven, supporting a probable causality. Liver function normalized after discontinuation of the supplement, underscoring the hepatotoxic potential of this widely used herbal agent [29].

Hepatic regeneration may induce fibrogenesis when injury is persistent or when normal repair mechanisms are overwhelmed. Transforming growth factor beta (TGF-β), released locally, activates hepatic stellate cells. These cells transdifferentiate from storage cells into proliferative myofibroblasts that secrete excessive extracellular matrixes, mainly collagen. Repeated cycles of injury and aberrant regeneration lead to progressive fibrotic tissue deposition, distortion of lobular architecture, and ultimately clinically manifest liver fibrosis.

In our reported case, clinical and biological evolution was favorable; nevertheless, the prolonged persistence of hepatocellular injury (3 months) resulted in a degree of hepatic fibrotic remodeling. At the last follow-up, 1 year and 7 months after the initial presentation (November 2025), hepatic evaluation by elastography and ultrasonography revealed a CAP of 253 dB/m (steatosis degree S0) and liver stiffness of 8.1 kPa, corresponding to Metavir stage 2 of fibrosis. Despite a clinically and biochemically stable status, these findings indicate a certain degree of hepatic fibrous matrix remodeling following the previous episode of intense hepatocellular injury.

## 4. Conclusions

Certain food-derived compounds that are common and readily accessible can, under specific individual conditions, act as potential toxic agents that are difficult to identify without a detailed history. Repeated administration in doses or concentrations exceeding usual culinary use may trigger idiosyncratic or directly toxic adverse reactions with significant clinical implications for liver function and overall health status. Any liver injury of unclear etiology, whether manifested solely by elevated liver enzyme levels or by cholestatic or mixed pattern, should raise suspicion of drug-induced or herb-induced liver injury. In such cases, systematic application of the updated RUCAM scoring system is essential, as it enables timely and reliable causality assessment, supports optimal patient management and, last but not least, refines diagnostic accuracy in liver diseases.

Given the rapidly expanding global use of dietary supplements, phytotherapeutics, and over-the-counter herbal formulations, we fully agree that drug- and herb-induced liver injury will represent a growing clinical and regulatory challenge in the coming decades. We therefore consider the promotion of the updated RUCAM not merely methodological but essential for future pharmacovigilance and clinical hepatology.

## Figures and Tables

**Table 1:** Laboratory tests with abnormal values at admission.

Parameter	Patient’s Value	Normal Range
Glycemia	119 mg/dL	74–100 mg/dL
HbA1 c	5.6	4–5.7%
C-reactive protein	3.2	0–5 mg/L
HDL cholesterol	39 mg/dL	40–60 mg/dL
LDL cholesterol	120.24	10–100 mg/dL
AST	196 U/L	5–34 U/L
ALT	357 U/L	0–55 U/L
GGT	14 U/L	9–36 U/L
Total bilirubin	0.66 mg/dL	0.2–1.2 mg/dL
Conjugated bilirubin	0.2 mg/dL	0–0.5 mg/dL
Alkaline phosphatase	92 U/L	40–150 U/L
International normalized ratio	1.01	INR
Activated partial thromboplastin time	28.9	25.1–36.5 s
Quick time	12	9.4–12.5 s
Prothrombin activity	94	70–140%

**Table 2:** Updated RUCAM criteria for HILI diagnostic support.

Nr.	Items for Hepatocellular Injury	Explanation	Score
1	Time to onset from start	Liver injury occurred approximately three months after starting the herbal product, a time frame compatible with drug-/herb-induced liver injury (5–90 days).	+2
2	ALT from cessation of the product (de-challenge)	Following cessation of the product, ALT continued to rise until day 8, reaching a peak of 637 U/L, followed by a plateau and subsequent progressive decrease, representing a positive de-challenge.	+2
3	Risk factors	The patient was 55 years old and did not consume alcohol; age represented a minor risk factor for hepatotoxicity.	+1
4	Concomitant drugs	The patient did not take any other hepatotoxic medications or supplements concomitantly with the herbal product.	0
5	Exclusions of other causes	All other possible causes of liver injury (viral hepatitis, alcohol, biliary disease, ischemia, and autoimmune disease) were excluded.	+2
6	Known hepatotoxicity	There was one reported case in the literature of hepatotoxicity associated with only this product.	+1
7	Re-exposure	The product was not readministered.	0
Total			Total score: 8 → Probable herb-induced liver injury

## Data Availability

The original contributions presented in the study are included in the article, further inquiries can be directed to the corresponding author.

## Abbreviations

The following abbreviations are used in this manuscript:

DILI	Drug-Induced Liver Injury
HILI	Herb-Induced Liver Injury
BMI	Body Mass Index
AST	Aspartate Aminotransferase
ALT	Alanine Aminotransferase
LDL	Low-Density Lipoprotein
HDL	High-Density Lipoprotein
CT	Computed Tomography
CAP	Controlled Attenuation Parameter
IgM	Immunoglobulin M
VHA	Virus Hepatitis A
HBsAg	Hepatitis B Surface Antigen
anti-HBc	Antibodies to Hepatitis B Core Antigen
IgG	Immunoglobulin G
HCV	Hepatitis C Virus
VHE	Virus Hepatitis E
AMA-M2	Anti-Mitochondrial Antibodies M2
ANA	Antinuclear Antibodies
AIH	Autoimmune Hepatitis
LKM-1	Liver-Kidney Microsomal Type 1 Antibodies
LC1	Liver Cytosol Type 1 Antibodies
SLA/LP	Soluble Liver Antigen/Liver Pancreas Antibodies
PML	Promyelocytic Leukemia Protein (Autoantibody Target)
gp210	Glycoprotein 210 Autoantibody
MAFLD	Metabolic Dysfunction-Associated Fatty Liver Disease
NAFLD	Non-Alcoholic Fatty Liver Disease
NASH	Non-Alcoholic Steatohepatitis
IARC	International Agency for Research on Cancer
updated RUCAM	Updated Roussel Uclaf Causality Assessment Method
DNA	Desoxyribonucleic Acid
